# FAMILY MATTERS: CROSS-CULTURAL DIFFERENCES IN FAMILISM AND CAREGIVING OUTCOMES

**DOI:** 10.1093/geroni/igad104.0443

**Published:** 2023-12-21

**Authors:** Francesca Falzarano, Jerad Moxley, Karl Pillemer, Sara Czaja

**Affiliations:** Weill Cornell Medicine, New York City, New York, United States; Weill Cornell Medicine, New York City, New York, United States; Cornell University, Ithaca, New York, United States; Weill Cornell Medicine, New York City, New York, United States

## Abstract

The increasing number of minority older adults and subsequent increase in minority individuals needed to provide care highlights the need to understand how the caregiving role is experienced and perceived among racial/ethnic minority groups. Familism, a cross-cultural collectivist value, has been examined as a driving mechanism influencing differential outcomes in family caregivers. However, research examining familism in caregiving has yielded mixed results. Guided by the Sociocultural Stress and Coping Model, the purpose of this study is to examine cross-cultural differences in familism and its influence on caregiving outcomes. Baseline data were collected from 243 family caregivers who participated in the Caring for the Caregiver Network randomized controlled trial, a culturally tailored technology-based psychosocial intervention. Results showed that familism was more highly endorsed by Hispanic and African American participants, when compared to Whites, which was subsequently associated with positive aspects of caregiving, depression, and burden. Exploratory analyses among the Hispanic subgroup were conducted to examine how acculturation, operationalized as number of years living in the US, influences familism and found that a longer length of time spent in the US was negatively associated with familism. When dividing our sample into U.S. natives versus non-natives, non-native Hispanic participants reported higher familism compared to natives. Findings indicate that cross-cultural differences in caregiving appraisals may operate as a function of cultural values, which influence one’s subjective evaluation of the caregiving role and contribute to more optimal outcomes – highlighting the need for culturally congruent interventions to promote positive caregiving outcomes particularly among racial/ethnic minorities.

